# Semantic organization in children with cochlear implants: computational analysis of verbal fluency

**DOI:** 10.3389/fpsyg.2013.00543

**Published:** 2013-09-02

**Authors:** Yoed N. Kenett, Deena Wechsler-Kashi, Dror Y. Kenett, Richard G. Schwartz, Eshel Ben-Jacob, Miriam Faust

**Affiliations:** ^1^The Leslie and Susan Gonda (Goldschmied) Multidisciplinary Brain Research Center, Bar-Ilan UniversityRamat-Gan, Israel; ^2^Department of Communication Sciences and Disorders, Ono Academic CollegeKiryat Ono, Israel; ^3^School of Physics and Astronomy, The Reymond and Beverly Sackler Faculty of Exact Sciences, Tel-Aviv UniversityTel-Aviv, Israel; ^4^Department of Physics, Center for Polymer Research, Boston UniversityBoston, MA, USA; ^5^Program in Speech-Language-Hearing Sciences, The Graduate Center, City University of New YorkNY, USA; ^6^Department of Psychology, Bar-Ilan UniversityRamat-Gan, Israel

**Keywords:** cochlear implants, semantic networks, verbal fluency, network science, spreading activation, mental lexicon

## Abstract

**Purpose:** Cochlear implants (CIs) enable children with severe and profound hearing impairments to perceive the sensation of sound sufficiently to permit oral language acquisition. So far, studies have focused mainly on technological improvements and general outcomes of implantation for speech perception and spoken language development. This study quantitatively explored the organization of the semantic networks of children with CIs in comparison to those of age-matched normal hearing (NH) peers.

**Method:** Twenty seven children with CIs and twenty seven age- and IQ-matched NH children ages 7–10 were tested on a timed animal verbal fluency task (*Name as many animals as you can*). The responses were analyzed using correlation and network methodologies. The structure of the animal category semantic network for both groups were extracted and compared.

**Results:** Children with CIs appeared to have a less-developed semantic network structure compared to age-matched NH peers. The average shortest path length (ASPL) and the network diameter measures were larger for the NH group compared to the CIs group. This difference was consistent for the analysis of networks derived from animal names generated by each group [sample-matched correlation networks (SMCN)] and for the networks derived from the common animal names generated by both groups [word-matched correlation networks (WMCN)].

**Conclusions:** The main difference between the semantic networks of children with CIs and NH lies in the network structure. The semantic network of children with CIs is under-developed compared to the semantic network of the age-matched NH children. We discuss the practical and clinical implications of our findings.

## Introduction

Cochlear Implantation (CI) has been the procedure of choice in the last quarter of a century for successful treatment of profound sensorineural hearing loss (Balkany et al., 2002). Over the years, approval for age of implantation has dramatically dropped, up to children in their first year of life (Peterson et al., 2010; Von Ilberg et al., 2011). Very young implantation brought expectations for children with CIs to develop language within normal-age limits. However, although early implantation generally yields good progress, many children with CIs still do not reach age equivalent language capabilities. In fact, this population shows great variance in their language performance, variance which is believed to result from factors that interact and effect language development trajectories in an unpredictable manner (Schwartz et al., 2013). Thus, it is difficult to predict children with CIs language development outcome (Geers et al., 2007). Several studies have demonstrated delays in lexical development in children with CIs post implantation (i.e., Blamey et al., 2001; Le Normand et al., 2003). For example, Walker and McGregor (2013) demonstrated deficits in word learning processes in a group of young early implanted children (ages of 3; 6–6; 9). Since It is important to gain insight into the specific characteristics of the diverse language aspects in children with CIs (Boons et al., 2012) and since recent findings suggest that toddlers' abilities to associate speech sounds with objects or to learn novel words are more strongly related to vocabulary development than to speech perception abilities (Schwartz et al., 2013), we examine the semantic organization of the lexicon in children with CIs. The research presented here is the first quantitative analysis of the semantic level of children with CIs, compared to that of age-matched normal hearing (NH) peers.

While most behavioral examination of language development in children with CIs post implantation point to high rates of improvement [using measures such as the Reynell Developmental Language Scales (Svirsky et al., 2000), Peabody Picture Vocabulary Test (PPVT; Dunn and Dunn, 1981, 1997), Clinical Evaluation of Language Fundamentals (CELF; Semel et al., 1995), Mean Length of Utterance (MLU; Blamey et al., 2001), and steeper rates of post operatives language measures (Dawson et al., 1995)], research indicates that many children with CIs may never reach full language capabilities as their NH peers (Peterson et al., 2010; Von Ilberg et al., 2011). The reasons for this are: limited auditory experience during the sensitive periods for normal language development, lack of audition in the period prior to implantation or limited auditory input provided by the implant (Peterson et al., 2010). Yet, much is unknown regarding the large variance in language development post implantation. For example, research conducted by Ouellet and colleagues analyzed the vocabulary exhibited by children with CIs implanted at the age range of 22–76 months (Ouellet et al., 2001; Le Normand et al., 2003). These authors found that children with CIs were delayed in vocabulary development compared to age matched NH peer norms, but did follow typical developmental stages of language acquisition. Furthermore, Geers et al. (2003) analyzed a large sample of children with CIs and found, in a battery of language tests, that only about half of the children in the study, which were expected to develop language within the normal range, showed language abilities comparable with that of hearing age mates.

While studies examining language in children with CIs using standardized, omnibus language tests provide important information on the effectiveness of implantation, they cannot explain why many children do not achieve age-appropriate language capabilities. It is unclear whether the poorer lexical abilities presented by some children with CIs are a result of poor lexical representation, or merely a by-product of their phonological deficit. In light of the lack of research on semantic development of CI subjects, such research is of value. In addition, examinination of the CI population offers a unique and unequaled opportunity to investigate processes of language development, due to the fact that these subjects present pure cases of incomplete language development that are related to auditory deprivation. As such, they provide a uniqiue opportunity to study many unanswered questions regarding language development.

Investigating the more specific mechanisms of language production in children with CIs, Wechsler-Kashi et al. (2013) examined lexical organization, using verbal fluency task in a group of implanted children and an age- and IQ- matched group of NH peers. Children with CIs generated significantly fewer words compared to the NH group on semantic (i.e., in 1 min, name all the *animals* you can think of) and phonological (i.e., in 1 min, name all words that start with the sound *f*) verbal fluency tasks. To date, there are no other reported studies referring specifically to the semantic level of processing or semantic representations in the CI population (see Schwartz et al., 2013 for more recent research). Wechsler-Kashi et al. (2013) show that children with CIs seem to access words in semantic and phonological categories less efficiently than NH peers. These differences were linked to differences in lexical representations, capacity or organization. These differences might derive from delays in lexical development (learning words for categories) or reflect phonological mediation on access to semantic information. Yet, the specific underlying mechanism responsible for these differences remains unknown. Is it a result of auditory issues, resulting in a smaller mental lexicon, or can the difference in lexical access of children with CIs shown by Wechsler-Kashi et al. (2013) be a result of deficits in the semantic memory level of this population? In order to address this issue, investigation of the semantic memory organization in children with CIs compared to NH peers is required. As Cleary (2009) suggested, this is an understudied, yet promising, area of research in children with hearing impairments.

Semantic memory is the system of human memory that stores concepts and facts, regardless of time or context. As a stricter definition, semantic memory is responsible for the storage of semantic categories and of natural and artificial concepts (Budson and Price, 2005; Patterson et al., 2007). How semantic memory is organized, and more specifically, which words are close to others and how this system is organized into subcategories, remains an open question (Rogers, 2008). Over the years, many different models have been suggested in attempt to model the organization of knowledge, yet to date, no one unifying model exists (see Rogers, 2008 for an extensive review). Recently, a growing amount of research in neurocognitive domains is being conducted through the use of computational network tools (graph theory based) mainly founded on the Small World Network model (SWN; Milgram, 1967; Watts and Strogatz, 1998). The use of such tools, based on the SWN model, in language research is developing and has already provided many unique insights into the nature of language processing and the organization of knowledge (Borge-Holthoefer and Arenas, 2010).

The basic components of a SWN are sub-clusters of nodes and relatively short path lengths (number of edges connecting two nodes in the network). Two main characteristics of SWNs are the networks clustering coefficient (CC) and its average shortest path length (ASPL). The CC refers to the probability that two neighbors (a neighbor is a node *j* that is connected through an edge to node *i*) of a randomly chosen node will themselves be neighbors. The ASPL refers to the average shortest amount of steps (nodes being traversed) needed to be taken between any two pair of random nodes. A SWN is characterized by having a large CC and a short ASPL. This model has successfully described a wide range of sociological, technological, biological and economical networks (Boccaletti et al., 2006; Cohen and Havlin, 2010; Kenett et al., 2010, 2012; Newman, 2010; Madi et al., 2011; Bransburg-Zabary et al., 2013). Furthermore, a rapidly growing body of neuroscientific research demonstrates and investigates the SWN features of the anatomical and functional levels of the brain (Sporns, 2011; Stam and van Straaten, 2012). The use of such network tools in cognitive domains is growing rapidly, mainly to investigate the complex system of language and memory structure (Vitevitch, 2008; Chan and Vitevitch, 2010; Vitevitch et al., 2012; see Baronchelli et al., 2013 for a recent review). In the linguistic domain, lexicons of different languages seem to display SWN characteristics, and is considered to be a fundamental principle in lexical organization. This type of organization allows for fast search and retrieval of information, thus capturing the core properties of semantic networks (Steyvers and Tenenbaum, 2005; Borge-Holthoefer and Arenas, 2010; Kenett et al., 2011).

Recently, the use of network tools in neurocognitive research has expanded into new fields, such as clinical populations and developmental research. The application of such tools in clinical populations, suffering from psychiatric or neuropsychological disorders, aims at examining such disorders via network tools, to provide quantitative observations that can be applied, empirically and clinically, to these populations (for a recent review, see Stam and van Straaten, 2012). Mota et al. (2012), for example, used a network based approach to study the speech produced by psychotic and schizophrenic patients, by creating “speech graphs” for each clinical population (Mota et al., 2012). They showed, for the first time, that a quantitative analysis of these speech graphs can differentiate between these two populations, providing valuable clinical information not measured by classical clinical measurements.

Applying such tools in developmental research made it possible to study the development of brain structure of children in their first years of life. The research has shown that as the brain develops, it re-organizes itself from a SWN state toward a more structured topology of brain networks (Boersma et al., 2011; Fan et al., 2011; Yap et al., 2011). For example, Boersma et al. (2011) made use of complex network tools to conduct a longitudinal examination of the development of EEG bands in resting state EEGs of 5-year-old children compared to 7-year-old children. The authors report an increase in CC and ASPL in all EEG bands measured, as children got older. This transition, from SWN toward structured networks, has also been demonstrated in an investigation of white matter pediatric development during the first year of life (Yap et al., 2011). From a cognitive perspective, network tools are being applied to examine language acquisition and new word learning mechanisms (Steyvers and Tenenbaum, 2005; Hills et al., 2009a,b). Hills et al. (2009a) applied network tools to investigate the developmental growth of early noun networks and examine the learning principles applied in such noun networks. As such, developmental computational research sheds new light on the nature of early brain organization, highlighting the importance of a transition from an initial SWN state in early brain networks toward a more structured, organized state.

As the amount of studies implementing network tools in clinical population research and the developing brain grow, research using computational tools to examine clinical issues of the developing brain is starting to emerge. Beckage et al. (2011) explored the difference in semantic networks of typically developing children and late-talking children. Although the semantic network of typically developing children exhibits SWN properties at as early as 15 months of age, late-talkers exhibit these SWN properties to a much lesser extent. As such, applying network tools to investigate the semantic organization of children with CIs may elucidate whether the poorer linguistic performance exhibited by children with CIs is related to their semantic network organization or it is purely phonological in nature. Specifically, can network tools shed further light on the performance of children with CIs in the verbal fluency research, conducted by Wechsler-Kashi et al. (2013)?

The verbal fluency task is widely used in neuropsychological and cognitive research (Ardila et al., 2006). In semantic verbal fluency tasks, subjects are required to generate words from a certain category (such as fruits or animals) in a certain amount of time (usually 60 s). While different semantic categories have been used for this task, the animal category is the most widely used, as it is more universal in its nature, with only minor differences found across different languages and cultures (Ardila et al., 2006). Although this task is easy to explain and conduct, it actually conveys a complex cognitive process. According to the main cognitive theory of the verbal fluency task, this cognitive process is comprised of two different processes—clustering and switching (Troyer et al., 1997, 1998; Troyer, 2000). Clustering refers to retrieving words within a subcategory. Switching refers to the process of switching from one subcategory, when the retrieval from this subcategory is exhausted, to a new subcategory. For example, in the animal category, clustering produces semantically related words (i.e., *dog*–*cat*) and switching allows jumping to a new animal subcategory (i.e., *cat*–*dolphin*) (see Troyer, 2000 for semantic subcategories norms).

While the verbal fluency task provides an efficient way to investigate semantic memory organization (easy task to conduct, yet provides rich information) scarce network research has so far been conducted on this task. This is due to the random nature of both the retrieval exhaustion of subcategories, and the switching process between subcategories. A pioneer research conducted by Goni et al. (2010b) developed a search algorithm in an attempt to model this process. Their algorithm is based on a random walk through a cluster of nodes, combined with switching to any other nodes with a certain probability, based on the Markov chains approach. Though the authors prove the feasibility of their approach, they acknowledge that their approach is not optimal. Currently there are two main studies attempting to computationally investigate the animal category organization expressed in this task. Lerner et al. (2009) investigated whether network analysis of the animal category fluency data can provide further evidence to the change in semantic organization between normal controls, persons with mild cognitive impairment and persons suffering from Alzheimer's disease. In their networks, nodes represented animal names generated by each of the three groups, and links between nodes were drawn simply based on whether these two nodes were named in succession by at least one subject. The results of this research revealed that the semantic network of the animal category of persons suffering from AD has a higher CC, shorter ASPL and a lower amount of low-frequency nodes. This work is the first network analysis of a verbal fluency task, supporting the fruitfulness of such research. Taking a more rigorous statistical approach, Goni et al. (2010a) analyzed the semantic organization of the animal category in a large sample of Spanish and English speaking subjects. In order to provide a statistical approach to determine link drawing between nodes, these authors developed a statistical framework based on the co-occurrence of animal words generated by their sample. While their method provides a more quantitative method to infer subcategory organization of the animal category, their method relies on a complex statistical method to predict links between nodes.

The present research introduces a new method to quantitatively analyze the verbal fluency task based on the correlation between word profiles generated by the sample. Our approach takes into account the individual random exhaustion of subcategories, based on subject responses. In this approach, we assume a general organization into subcategories, even if not completely exhausted by an individual subject. For example, if one subcategory of the animal category is household pets, includes *cat, dog*, etc., than when retrieving from this subcategory the word *dog*, it is likely that the word *cat* will also be retrieved. Thus, we expect a high correlation score between the appearance of the word *dog* and the appearance of the word *cat* in our sample—participants who generate the animal word *dog*, are likely to also generate the animal word *cat*.

In the present study we conduct the first quantitative research on the semantic network of children with CIs, by quantitatively comparing the semantic network of these children with the semantic network of their normal age-matched hearing peers. To this end, we analyzed the results of a verbal fluency task (Wechsler-Kashi et al., 2013), in order to construct the semantic networks of both CI and NH groups. Analyzing the properties of these semantic networks allowed us to identify and quantify the difference between these two groups.

## Method

The data analyzed in this research was gathered by Wechsler-Kashi et al. (2013). In the present research we analyzed only the *animal* category task of the VF experiment. In this task, subjects had to generate as many names of animals they could think of, in 1 min. Their responses were recorded and later on analyzed on various parameters (Wechsler-Kashi et al. (2013) for a complete description).

### Participants

Fifty-four subjects aged 7–10 were included in the analysis. All participants underwent language screening via the Clinical Evaluation of Language Fundamentals (CELF-3; Semel et al., 1995). In addition, the Test of Non-verbal Intelligence (TONI 3; Brown et al., 1997) was administered to NH and CI subjects. Only NH children who passed both a hearing screening (described below) and the CELF language screening were included. All children included in the analysis had normal IQ scores (above 80 on TONI non-verbal intelligence test). English was the native and primary language of all children tested.

Twenty-seven NH children (ages 7; 0–10; 8, *M* = 8; 9, *SD* = 1.12) were included. Seventeen were girls and 10 were boys. Their average TONI score was 109.3 (*SD* = 10.2). The inclusion criteria for hearing participants were: no parentally reported history of speech or language deficits, no reported neurological or emotional disorders and no known visual impairments that cannot be corrected by glasses. All hearing children underwent an audiological screening before initiating the actual experiments. This screening was conducted at 20 dB in frequencies 500, 1000, 2000, and 4000 Hz. Two responses were required in each frequency in each ear in order to pass the hearing screening.

Twenty-seven children with CIs (ages 7; 0–10; 8, *M* = 8; 9, *SD* = 1.11) were included. Eleven were girls and 16 were boys. Their average TONI score was 109.7 (*SD* = 11.5). CI participants had severe to profound bilateral sensori-neural hearing loss diagnosed before the age of 3 years, with at least 6 months of experience with CI prior to participation in this study. Nine children used one CI device in one ear only (right or left), 10 had bilateral implants, and 8 had a combination of a CI in one ear and a hearing aid in the other (bi-modal amplification). None of the children included in the analysis had a history of infections or device failure that had caused non-use of the CI for a long period of time. All the children in the CI group were users of oral communication or total communication. The children with CIs were asked to use the typical setting for their CI(s) and hearing aid.

### Data preparation

In order to analyze the data, we converted the responses for each group into data matrices. This matrix was constructed such that each row contains all answers of a single (CI or NH) subject, and each column is a unique animal name given by the entire sample. Each cell, therefore, denotes whether a subject *i* generated animal word *j* or not, in a binary manner— “1” indicating that subject *i* generated animal noun *j*, and a “0” indicating that he/she did not. The NH participants, as a group, named 132 different animal names, and the CI participants named 106 different animal names. In this sense, the analysis resulted in a 27 (subjects) × 132 (animal words) matrix for the NH group and a 27(subjects) × 106 (animal words) matrix for the CI group.

### Word correlations

The first step in constructing the semantic networks was calculating correlations between the responses to the verbal fluency task. These correlations convey the relation between two word profiles (the responses of a specific word by all subjects; e.g., *cat* and *dog*), and were calculated by Pearson's formula:

(1) $$C(i,j)=\frac{\left(X_{i}-\mu_{i})(X_{j}-\mu_{j}\right)}{\sigma_{i}\sigma_{j}}$$

Where *x*_*i*_ and *x*_*j*_ are the response profile of words *i* and *j*, and σ_*i*_ and σ_*j*_ are the STD of the response profiles of words *i* and *j*. Note that the word-word correlations (or in short, word correlations) for all pairs of words define a symmetrical matrix, in which each (*i, j*) element is the correlation between words *i* and *j*.

### Correlation based semantic networks

The word correlation matrix can be studied in terms of an adjacency matrix of a weighted, undirected network. In this approach, each word is a node in the network, and an edge (link) between two nodes (words) is the correlation between them, with the correlation value being the weight of that link. Thus, the word correlation matrix represents a fully connected weighted network in which nodes represent words, and links represent the correlations between words. This representation can be filtered to uncover the most meaningful information about the network. Here we used the Planar Maximally Filtered Graph (PMFG) method to this end (Tumminello et al., 2005; Kenett et al., 2011). Since we were primarily interested in the structure of the networks, we binarized the networks (by converting all edges to a uniform weight = 1) and analyzed the networks as unweighted, undirected networks.

Because these word correlation networks are based upon matched sample size for both groups—children with CIs and NH children, we define these word correlation networks as Sample-Matched Correlation Networks (SMCN). In order to remove any sample size bias, we also added a second analysis, in which we analyzed data from both groups, based on common target animal words given by both groups, by at least two subjects in each group. This resulted in 75 common animal words, generated by both groups. We define these networks as Word-Matched Correlation Networks (WMCN). This process enabled us to quantitatively compare the differences between the networks.

The network parameter calculations (for both SMCNs and WMCNs) were performed with the Brain Connectivity Toolbox for Matlab (Rubinov and Sporns, 2010). The network parameters calculated were the CC, the ASPL (L), the network's diameter (D), and the mean degree number (<k>) (Boccaletti et al., 2006). To examine the network's CC and ASPL, a random network was created with the same number of nodes and edges. The clustering coefficient (CCrand) and ASPL (Lrand) of these random networks were calculated. Finally, the small-world-ness measure (S; Humphries and Gurney, 2008) was calculated to quantitatively examine the small-world nature of the network. This measure examines the trade-off between the networks CC and its ASPL and is the first quantitative measure established for quantifying the “small-world-ness” feature of a specific network. Using this measure, any *S*-value larger than 1 (*S* > 1) defines the network as a SWN.

Finally, the WMCNs allow us to quantitatively compare the CI and NH networks. To this end, we make use of the word-centrality concept (Kenett et al., 2011) to quantify the importance of each word (node) in the network. Thus, in the network, a word impact score of node *i* is defined as the difference between the ASPL of the network when removing word *i*, with the ASPL of the full network:

(2) WC(i)=〈SP(A∉i)〉−〈SP(A)〉

Where *A* represents the network adjacency matrix, and 〈SP〉 is the ASPL of the network. Hence, this impact score indicates the centrality, or importance, of a word *i* in the network.

## Results

### Sample-matched correlation networks

We began the analysis by constructing the SMCN from the word correlation matrix, using the PMFG filtering process. Then, the different SWN properties of the SMCNs were calculated (Table 1).

**Table 1:** **Summary of results of sample-matched correlation networks analysis**.

**Parameter**	**NH**	**CI**
N	132	106
L	6.15	4.69
D	16	11
CC	0.66	0.67
< k >	5.91	5.89
CCrand	0.05	0.05
Lrand	2.93	2.76
S	6.57	7.83

N, number of nodes in the network; L, average shortest path length; D, diameter; CC, clustering coefficient; < k >, mean degree; CCrand, clustering coefficient of random graph; Lrand, average shortest path length of random graph; S, small-world-ness measure.

**Figure 1:**
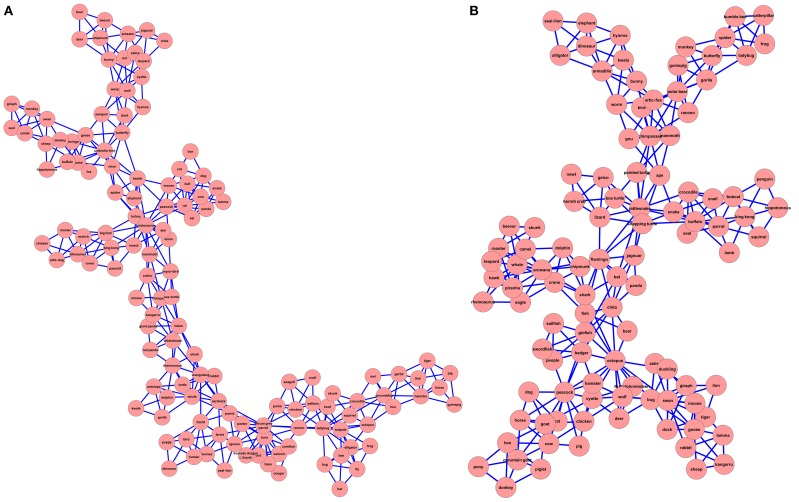
**2D visualization of Sample-Matched correlation networks for the NH group (A) and the CI group (B)**.

These results show that both SMCNs are SWNs. The CC for both networks was much higher than those of their matched random networks. Furthermore, the small-world-ness measure also indicated that both networks are SWN (*S* > 1). The results reveal that the NH-SMCN is more spread out than the CI-SMCN: The NH-SMCN diameter is larger than that of the CI-SMCN (*D*_*NH*_ = 16 > *D*_*CI*_ = 11); the CC of the NH-SMCN is lower than that of the CI-SMCN, indicating that the CI-SMCN is more condensed than the NH-SMCN (*CC*_*NH*_ = 0.66 < *CC*_*CI*_ = 0.67) and the ASPL of the NH-SMCN which is larger than that of the CI-SMCN ASPL, indicating that the NH-SMCN is more disperse than the CI-SMCN (*L*_*NH*_ = 6.15 > *L*_*CI*_ = 4.69). Finally, while the CI-SMCN has fewer nodes than the NH-SMCN (106 nodes vs. 132 nodes), it has a larger small-world-ness score (*S*_*NH*_ = 6.57 < *S*_*CI*_ = 7.83). This difference in network structure is further evident in a visual presentation of the SMCNs (Figure 1), which was done using the Cytoscape software (Shannon et al., 2003). Altogether, by visually and quantitatively examining the underlying representation of both SMC networks, it is apparent that there is a difference in structure, with the NH-SMCN being more spread out than the CI-SMCN.

This difference between the two networks might be affected by the difference in size of the two network (106 nodes for the CI-SMCN, compared to 132 nodes for the NH-SMCN) or by the different words constituting the two networks (as both group's generated somewhat different animal words). In order to eliminate these possible confounds, and in order to compare the semantic networks of both groups, we constructed a WMCN for each group. These WMC networks are comprised of only the common words generated by both groups (75 words). If the semantic networks of both groups are similar, we do not expect any differences in their semantic network statistics.

### Word-matched correlation networks

**Figure 2:**
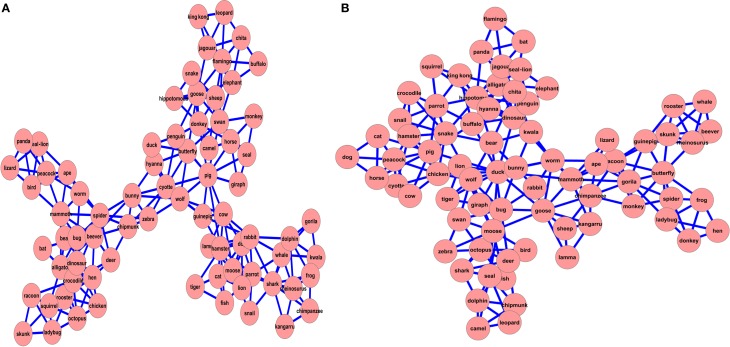
**2D visualization of word-match correlation networks for the NH group (A) and the CI group (B)**.

We calculated both WMCNs, in the same method described above. We then calculated the different SWN measures of the WMCNs (Table 2). The results show that both WMCNs are SWN. The CC for both networks is much higher than that of their matched random networks. In addition, the small-world-ness measure indicates that both networks have SWN characteristics. If both networks were similar, we would not expect any differences in their SWN parameters, which is clearly not the case. Similar to our findings for the SMC networks analysis, the two groups seem to differ in their network structure. This can be seen visually (Figure 2), and is also supported quantitatively by the parameters measured (Table 2). Results of WMC networks analysis indicate that the two groups differ in their diameter and ASPL, indicating that the NH-WMC network is more spread out than the CI-WMC network, thus replicating the findings of the SMCNs analysis.

**Table 2:** **Summary of results of word matched correlation networks analysis**.

**Parameter**	**NH**	**CI**
N	75	75
L	4.30	3.52
D	9	7
CC	0.67	0.65
< k >	5.84	5.84
CCrand	0.06	0.06
Lrand	2.59	2.57
S	6.80	7.06

N, number of nodes in the network; L, average shortest path length; D, diameter; CC, clustering coefficient; < k >, mean degree; CCrand, clustering coefficient of random graph; Lrand, average shortest path length of random graph; S, small-world-ness measure.

By calculating the WMC networks that are composed of the same words generated by both groups, we can further examine the difference between the semantic networks of both groups. This was done by three different statistical analyses: first, we generated a large sample of random networks with the same number of nodes and edge probability between nodes as in the WMC networks. This was done to examine whether the network measures calculated for the WMCNs do not result from a null-hypothesis random network. Second, we used the bootstrap method (Efron, 1979) to generate partial WMCNs and examined the difference between the network measures distribution between these two bootstrapped partial WMCNs. This was done in order to examine whether the statistical measures computed for the WMCNs reliably signify marked differences between the two networks. Finally, we measured word centrality—calculating the impact score of each node (word) in the network for both WMC networks and then compared the results of each group.

### Network validation

To validate the topological properties of the networks, and more important, the topological differences between the NH and CI groups, we use two complementing validation methodologies:

#### Simulation of random networks

Here we examine the statistical measures calculated for both WMC networks, and test that they do not result from a null-hypothesis of a random network. To this end, we generate a large sample of random networks with a fixed edge probability and compare the WMCN topological measures to the values resulting from the bootstrapped simulations.

To conduct this analysis, we first generated an Erdos-Renyi (Boccaletti et al., 2006; Cohen and Havlin, 2010) random network, with the same number of nodes as of the WMCNs, and with the same probability for an edge to be drawn between two nodes. For this random network, the three main network measures (CC, D, L) were calculated. This process was simulated with 10,000 realizations of the network, and resulted with a reference bootstrapped distribution for each network measure. The empirical values are then compared against their reference distribution to evaluate their statistical significance. This analysis revealed that for both of the WMC networks, all three network measures were statistically significant (all *p*'s < 0.001; Table 3).

**Table 3:** **Summary of results for simulated random network distribution analysis**.

**Parameter**	**NH**	**CI**	**SRN**
L	4.3	3.52	2.58 (0.08)
D	9	7	5 (0.5)
CC	0.67	0.65	0.08 (0.01)

L, average shortest path length; D, diameter; CC, clustering coefficient; NH, NH-WMC network; CI, CI-WMC network; SRN, simulated random network.

#### Bootstrapped partial WMCNs

To examine whether the statistical measures computed for both networks reliably signify a marked difference between them, we made use of the bootstrap method (Efron, 1979) to simulate random partial WCMN networks for both NH and CI groups and compared these networks. This was done for two reasons: (1) if the two WMC networks truly differ from each other, then any sub-network consisting of the same nodes in both networks should also be different and (2) the bootstrap method enables the generation of many simulated partial WMC networks, allowing us to statistically examine the difference between the two networks.

In order to conduct our statistical analysis of bootstrapped partial WMC networks, we randomly chose 40 words (nodes) out of the entire 75 words comprising the WMCNs, then constructed for each group separately their partial WMCNs of these random 40 words, and for each such partial WMCN computed their L, D, and CC measures. This process was simulated with 10,000 realizations, and resulted in 10,000 samples of partial WMC networks for groups and their L, D, and CC measures. An independent sample *t*-test analysis was conducted to investigate for each network measure the difference between both bootstrapped partial WMC networks (Table 4). This analysis revealed a significant difference between the two distributions for all three network measures (all *p*'s < 0.001). Thus, while these differences are numerically small, they are significantly different and replicate the main findings—the diameter and the ASPL of the NH network is larger than that of the CI network.

**Table 4:** **Summary of results of bootstrapped partial word matched correlation networks analysis**.

**Parameter**	**NH**	**CI**
L	2.96 (0.24)	2.94 (0.24)
D	3.60 (0.57)	3.56 (0.57)
CC	0.667 (0.02)	0.668 (0.02)

L, average shortest path length; D, diameter; CC, clustering coefficient; NH, partial NH-WMCN distribution; CI, partial CI-WMCN distribution. Values represent distribution mean value. The difference between L, D, and CC measures for both partial networks is significantly different (all p's < 0.001).

### Word centrality

The search for words that have a significant importance in a semantic network can be conducted with network tools. In network theory, the importance of a node is quantified using different measures, such as the betweeness-centrality or the eigenvalues centrality measures (Boccaletti et al., 2006). Here we used the word centrality measure (Kenett et al., 2011). This measure quantifies the impact of a certain node *i* on the network, defined as the difference between the ASPL of the network after removing node *i* with the ASPL of the full network.

In order to investigate the impact of a given word *i* in both WMC networks, we iteratively removed each word from the sample, and recalculated the WMC network and the ASPL of the network without this specific word, and then calculated the impact of each of the 75 words, as defined above (Kenett et al., 2011). This was performed separately for both WMC networks. As the path length measure is directly related to the spread of activation within the network (Balota and Lorch, 1986; De-Groot, 1989), calculating the impact score of every node measures the nodes general effect on the spread of activation within the network. A positive impact score signifies that after the deletion of word *i*, the ASPL became larger than the average ASPL of the full network, indicating that this word has a positive effect on the spread of activation within the network. In contrast, a negative impact score signifies that after the deletion of word *i*, the ASPL became smaller than the ASPL of the general network, indicating that this word has a negative effect on the spread of activation within the network.

**Figure 3:**
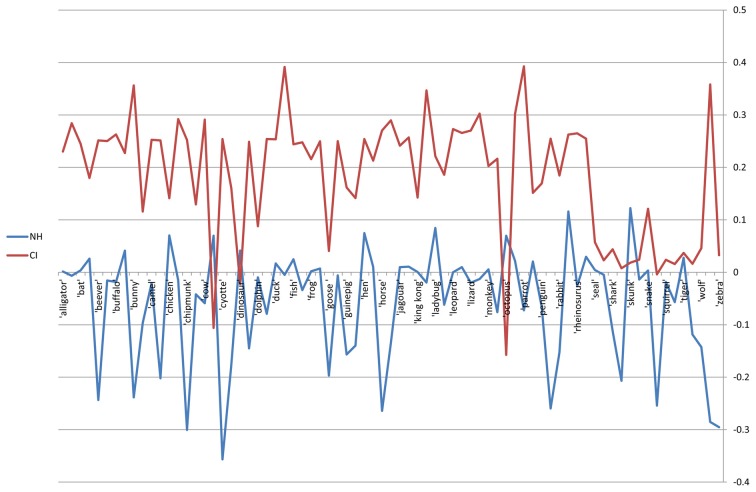
**Word impact score for both word-matched correlation networks**. The NH network is denoted in blue, and the CI network is denoted in red. *X*-axis—75 word-matched nodes. *Y*-axis—centrality score.

If the networks do not differ, removal of a word *i* from both networks should have a similar effect on the ASPL of the networks. As such, further than providing the effect each node has on the spread of activation in the network, calculating the impact score of the nodes for both networks allows us to statistically examine whether the networks differ. The impact score of all nodes in the WMCNs are plotted in Figure 3. A two-sample independent *t*-test between the impact scores of CI and NH groups revealed a significant difference between them [*t*_(148)_ = 13.218; *p* < 0.01]. As all words in the WMCNs were generated by both groups, this difference indicates a difference in the general structure of the network. This significant difference between the impact scores for all nodes between the two groups further strengthens the findings of the quantitative analysis that indicates a difference in the structure of the network between the two groups.

## Discussion

In this paper we present the first quantitative examination of the semantic network of children with CIs compared to the semantic network of age-matched NH children. Since research on the semantic aspects of children with CIs is scarce (Schwartz et al., 2013; Wechsler-Kashi et al., 2013), the work presented here takes a step forward in understanding the nature of the semantic organization in children with CIs.

We quantitatively analyzed the responses given by a sample of children with CIs and a matched control group of NH children to a semantic verbal fluency task of the animal category (Wechsler-Kashi et al., 2013). These responses were used to construct for each group their word correlation matrix—a matrix donating the similarities of response profiles between animal words generated by each group seperately. The word correlation matrices were then used to extract the semantic networks of children with CIs and their age-matched NH peers. This was done by constructing for both groups word correlation networks either matched by sample size (SMCN) or by words generated by both groups (WMCNs). These networks allowed us to investigate the characteristics and structure of each network on its own, and more importantly to compare between the two networks (for the WMCNs).

The results demonstrate the SWN nature of both CI and NH semantic networks. However, two main differences are consistent across the analysis of both types of networks: first, the diameter of the network is larger for the NH compared to the CI network; and second, the ASPL is larger for the NH compared to the CI network. These differences were consistent for both the SMCN, which analyzed the entire words generated by each group, and for the WMCNs, which analyzed only words that were retrieved by both groups. Thus, the main difference between the semantic networks of children with CIs compared to their age-matched NH peers seems to lie in the structure of the network—the semantic network of children with CIs is more condensed and less spread out than the NH semantic network.

Furthermore, in order to statistically investigate the difference between the WMC networks, we conducted a bootstrap analysis on the networks. First, we generated a large sample of random networks with the same number of nodes and probability of an edge between nodes as those of the WMCNs. For these random networks we calculated the network topological measures and reiterated this process 10,000 times. This process resulted in a reference distribution of the values of the different topological measures investigated. We then examined the statistical significance of either of the WMC networks topological measures to fall within their bootstrapped reference distribution. This analysis revealed that all WMCNs measures are significantly outside the reference distribution, marking that both WMCNs are not a result of a hull-hypothesis random network. Next, we used the bootstrap method to investigate whether the two WMCNs significantly differ from each other. This was done by randomly creating partial WMC networks, calculating network measures for these partial WMC networks and reiterating this process 10,000 times. We then statistically compared the bootstrap distribution of the network measures for both WMC networks. This analysis found significant differences between all bootstrapped measure distribution, providing further evidence for the differences between the two groups, in the sense that both ASPL and diameter were significantly larger for the bootstrapped partial NH WMC network compared to the CI WMC network.

Finally, our calculation of impact score for each node in both WMC networks allowed us to examine how each node (word) influences the spread of activation within the network and to compare these effects between both networks. This examination revealed a significant difference between both networks. If the networks were the same, the removal of a random node *i* would have none or similar effect on both networks, contrary to what we found. While the NH-WMCN contains mostly positive impact score nodes, thus indicating that most nodes contribute in part to the spread of activation within the network, the CI-WMCN contains mostly negative impact score nodes, thus indicating that most nodes inhibit the spread of activation within the network. Thus, this impact score calculation strengthens our argument regarding the key factor differentiating CI and NH semantic networks—the structure of the network. A possible implication of the differences in the ease of spreading activation between the two groups may account for the difficulties of children with CIs in processing relevant aspects of semantics which require the activation of multiple (including distantly related) meanings (i.e., Kenett et al., 2011). For example, the development of types of language capabilities that require access to multiple meanings, including more distantly related meanings (e.g., ambiguous, metaphoric and figurative language) may depend on the structure of the network. As such, we postulate that the CI semantic network structure characteristics uncovered in this research may influence further higher-order linguistic abilities, which we plan to further study in future work.

Current studies using computational tools to examine the developing brain have revealed a transition in the nature of brain networks, from a SWN to a more ordered, structured nature (Boersma et al., 2011; Fan et al., 2011; Yap et al., 2011). In the semantic domain, Beckage et al. (2011) have shown that late-talking children exhibit a less Small World semantic network than typical-talking children. Taken together, it seems that a similar transition from a SWN to a more ordered network is crucial for normal language development (Boersma et al., 2011; Fan et al., 2011; Yap et al., 2011). Our findings show that children with CIs exhibit a less ordered semantic network than age- and IQ- matched NH peers. We interpret this less ordered state to imply a lag in the natural network transition, which might explain the various semantic difficulties exhibited by this population. In this sense, children with CIs exhibit a semantic network which might be similar to younger children rather than to their age-matched NH peers. Thus, developing clinical applications to facilitate transition of their semantic network from its small-world state to a more structured state may enhance their semantic capabilities.

The current study demonstrates the feasibility and importance of applying network tools to examine semantic organization in the CI population. While the results presented here derive only from the *animal* semantic category, it would be fruitful to analyze additional semantic categories in order to draw firm conclusions regarding differences in semantic organization of CI and NH children. We plan to further expand our network analysis of the CI children data collected by Wechsler-Kashi et al. (2013), and to apply further advanced quantitative measures on this data, both for the semantic and phonological categories. Furthermore, we plan to conduct additional qualitative analyses on this data to expand the quantitative findings presented here. Finally, we plan to collect similar data from a sample of younger NH children, to verify our results and provide further evidence for lexicon organization development. This sample of younger children will be matched in lexicon size and allow us to better examine the nature of the lag in lexicon development we uncovered in children with CIs (see Rice, 2003).

In summary, the work presented here is the first quantitative research examining the semantic network of children with CIs. We compared the semantic network of children with CIs to that of age matched NH peers. This comparison allowed us to quantitatively show the difference between these two semantic networks and to describe the nature of this difference. While further work is required in the area of semantics in children with CIs, insights from this and future research might enhance children with CIs semantic processing abilities through therapy, and may shed light on processes related to language acquisition.

### Conflict of interest statement

The authors declare that the research was conducted in the absence of any commercial or financial relationships that could be construed as a potential conflict of interest.
